# Synthesis and Characterization of Tetrakis(pentafluoroethyl)aluminate

**DOI:** 10.1002/chem.202000668

**Published:** 2020-09-17

**Authors:** Natalia Tiessen, Beate Neumann, Hans‐Georg Stammler, Berthold Hoge

**Affiliations:** ^1^ Universität Bielefeld Fakultät für Chemie Centrum für Molekulare Materialien Universitätsstraße 25 33615 Bielefeld Germany

**Keywords:** aluminum, perfluoroalkyl, phosphazenium cation, silane, weakly coordinating anions

## Abstract

While perfluorinated aryl, aryloxy and alkoxy aluminum species are well‐established as weakly coordinating anions (WCAs), corresponding perfluoroalkyl aluminum derivatives are virtually unknown. Reaction of Si(C_2_F_5_)_3_CH_3_ with Li[AlH_4_] afforded the tetrakis(pentafluoroethyl)aluminate, [Al(C_2_F_5_)_4_]^−^. Several salts of the [Al(C_2_F_5_)_4_]^−^ ion were synthesized and characterized by NMR spectroscopic methods, mass spectrometry, X‐ray diffraction studies and elemental analysis.

## Graphical abstract

**As perfluoroalkyl‐substituted aluminum compounds** are virtually unknown, this work presents a convenient synthesis of a tetrakis(perfluoroalkyl) aluminum species, the tetrakis(pentafluoroethyl)aluminate, [Al(C_2_F_5_)_4_]^−^. Several salts of the [Al(C_2_F_5_)_4_]^−^ ion are accessible by treatment of Li[AlH_4_] with Si(C_2_F_5_)_3_CH_3_ and subsequent cation exchange.

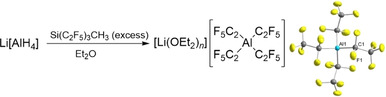

Several tetrakis(perfluoroaryl) and ‐(alkyl) derivatives of group 13 elements B, Al and Ga are known to date. A wide variety of these compounds are functioning as very efficient and popular weakly coordinating anions (WCAs) and have found application in room temperature ionic liquids (RTILs), as electrolytes for lithium‐ion batteries or in catalytic processes.[^1^, ^2^]

Probably the most popular perfluoroaryl substituted WCA is the commercially available tetrakis(pentafluorophenyl)borate, [B(C_6_F_5_)_4_]^−^, which is used in catalysis.[^3^, ^4^] Although the corresponding gallate [Ga(C_6_F_5_)_4_]^−[5]^ and the aluminate [Al(C_6_F_5_)_4_]^−[6]^ tend to decompose, they are employed in olefin polymerization processes.[^4^, ^7^]

Particularly in aluminum chemistry the alkoxy (OR^F^) and aryloxy (OAr^F^) substituted WCAs [Al(OR^F^)_4_]^−^ and [Al(OAr^F^)_4_]^−^ are highly prominent due to their stability and facile synthesis from Li[AlH_4_] and the corresponding alcohols.[^2^, ^8^] The resulting lithium salts are suitable for various metathesis reactions. Several boron analogues were also synthesized and are possibly useful as electrolytes in lithium‐ion batteries.[^2^, ^9^]

A further category of WCAs is based upon group 13 pentafluorotellurates (teflates). While [B(OTeF_5_)_4_]^−^ is known since 1981,^[10]^ the higher homologues [Al(OTeF_5_)_4_]^−[11]^ and [Ga(OTeF_5_)_4_]^−[12]^ have only recently been synthesized and have led to a considerable extension of WCA chemistry.

The tetrakis(trifluoromethyl)borate anion, [B(CF_3_)_4_]^−^, which is accessible through fluorination of [B(CN)_4_]^−^ with ClF_3_, is a surprisingly stable WCA that can be handled even in aHF.^[13]^ The analogous [Ga(CF_3_)_4_]^−^ was generated in 1991 and characterized by ^19^F NMR spectroscopy.^[14]^ Recently we reported on the tetrakis(pentafluoroethyl)gallate, [Ga(C_2_F_5_)_4_]^−^, which features a comparable stability to [B(CF_3_)_4_]^−^.^[15]^ Aluminum perfluoroalkyls however are virtually unknown. So far, a few perfluoropropyl aluminum species could be detected by ^19^F NMR spectroscopy but due to their instability they could not be isolated.^[16]^ In addition, numerous computational studies were performed on the Lewis acidity of Al(CF_3_)_3_ whereby, to the best of our knowledge, neither Al(CF_3_)_3_ nor its corresponding anion [Al(CF_3_)_4_]^−^ are known to date.^[17]^

Due to their tendency to eliminate difluorocarbene, trifluoromethylated inorganic compounds with E−CF_3_ bonds (with E=B,^[18]^ Si,[^19^, ^20^] Sn,^[21]^ Ge,^[22]^ P,^[23]^ or Cd^[24]^) generally exhibit a limited thermal and chemical stability. In contrast to this, their pentafluoroethyl analogues are remarkably more stable.^[25]^ This increased stability already becomes perceivable by the comparison of trifluoromethyllithium, LiCF_3_, and pentafluoroethyllithium, LiC_2_F_5_. While LiCF_3_ decomposes even at −78 °C,[^26^, ^27^] the corresponding LiC_2_F_5_ is stable up to −40 °C and therefore serves as a well‐established transfer reagent for pentafluoroethyl groups.^[28]^ With regard to this, the pentafluoroethyl group appears to be a promising candidate for the synthesis of the yet unknown aluminum tetrakis(perfluoroalkyls). Herein we give an account on the synthesis and molecular structure of the tetrakis(pentafluoroethyl)aluminate [Al(C_2_F_5_)_4_]^−^.

As demonstrated by the Finze group in a parallel study, the reaction of aluminumtrichloride, AlCl_3_, with LiC_2_F_5_ leads to the generation of the tetrakis(pentafluoroethyl)aluminate ion, [Al(C_2_F_5_)_4_]^−^.^[29]^

During our investigations of hydridosilicates we encountered a very efficient synthesis by accident. With the aim to generate the hydridosilicate [Si(C_2_F_5_)_3_H_2_]^−^, the corresponding silane Si(C_2_F_5_)_3_H was treated with Li[AlH_4_]. Here instead of the anticipated silicate, the tetrakis(pentafluoroethyl)aluminate ion, [Al(C_2_F_5_)_4_]^−^, was formed.

Since the synthesis of Si(C_2_F_5_)_3_H is elaborate,^[30]^ the more readily accessible Si(C_2_F_5_)_3_CH_3_ was utilized for further investigations. Si(C_2_F_5_)_3_CH_3_ results from pentafluoroethylation of SiCl_3_(CH_3_) with LiC_2_F_5_ in one step.^[20]^ When four equivalents of Si(C_2_F_5_)_3_CH_3_ are treated with Li[AlH_4_], the formation of the tetrakis(pentafluoroethyl)aluminate ion, [Al(C_2_F_5_)_4_]^−^, occurs immediately (Scheme 1).

**Scheme 1:**
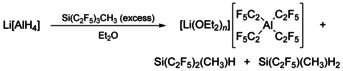
Generation of [Li(OEt_2_)_*n*_][Al(C_2_F_5_)_4_] from Li[AlH_4_] and Si(C_2_F_5_)_3_CH_3_.

In addition, hydridosilanes are identified in the reaction mixture. By analogy to the Ruppert–Prakash trifluoromethylation reaction,[^26^, ^31^] it is conceivable that a hydride ion adds to Si(C_2_F_5_)_3_CH_3_, thus activating a Si−C_2_F_5_ bond with subsequent transfer of the pentafluoroethyl group to aluminum (Scheme 2).

**Scheme 2:**
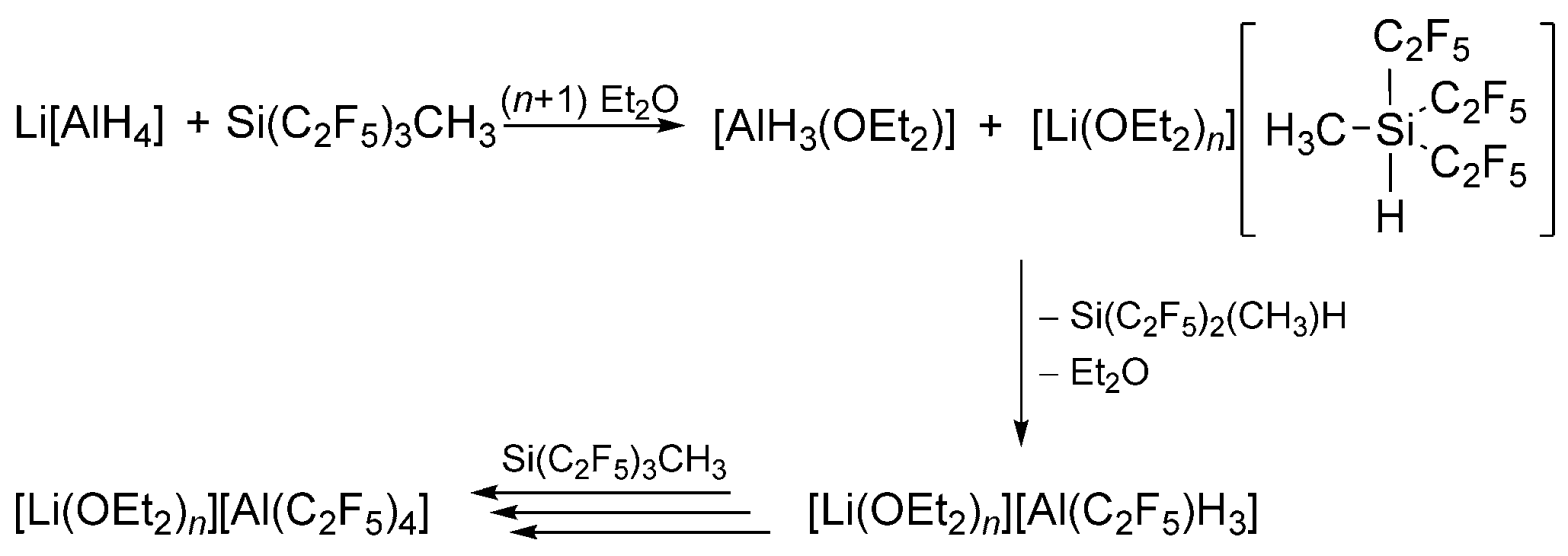
Formation of [Al(C_2_F_5_)_4_]^−^ via the activation of Si(C_2_F_5_)_3_CH_3_ by Li[AlH_4_].

Due to the fast rate of this transfer, spectroscopic proof of neither pentafluoroethyl‐hydrido aluminates nor silicates as intermediates in the reaction mixture was possible. Removal of all volatile components gives a colorless, extremely sensitive solid that slowly decomposes within days at room temperature. A cation exchange with [PPh_4_]Cl, [NBu_4_]Cl and [PNP]Cl (PNP=bis(triphenylphosphine)iminium) affords the corresponding salts. While [PPh_4_][Al(C_2_F_5_)_4_] and [NBu_4_][Al(C_2_F_5_)_4_] slowly decompose at room temperature, the corresponding PNP salt exhibits an increased thermal stability.

Weakly coordinating phosphazenium cations, as originally introduced by Schwesinger et al.,^[32]^ are outstanding in stabilizing reactive anions like for example the hydroxide trihydrate anion [OH(OH_2_)_3_]^−^.^[33]^ Consequently we employed [{(Et_2_N)_3_P=N}_3_PN(H)*t*Bu]Cl ([EtP_4_H]Cl) for a cation exchange to obtain [EtP_4_H][Al(C_2_F_5_)_4_] in an overall yield of 85 % (Scheme 3). The salt decomposes above 110 °C. This thermal stability of [EtP_4_H][Al(C_2_F_5_)_4_] allowed a characterization of the [Al(C_2_F_5_)_4_]^−^ anion by elemental analysis (found for C_48_H_100_AlF_20_N_13_P_4_: 41.59 % C, 7.56 % H and 13.33 % N; calcd 41.47 % C, 7.25 % H, 13.10 % N) underlining the identity and purity of the salt.

**Scheme 3:**
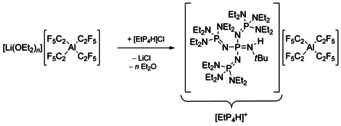
Synthesis of [EtP_4_H][Al(C_2_F_5_)_4_] by cation exchange.

In the ^27^Al NMR spectrum (Figure 1) the resonance of the [Al(C_2_F_5_)_4_]^−^ ion is observed as a nonet at *δ*=107.7 ppm with a ^2^ *J*(^27^Al, ^19^F) coupling constant of 32 Hz. In the ^19^F NMR spectrum a singlet at *δ*=−83.5 ppm for the CF_3_ groups is observed. Due to the nuclear spin quantum number of *I*=5/2 for aluminum, the signal for the CF_2_ units appears as a six‐line multiplet at *δ*=−127.7 ppm (Figure 2).

**Figure 1:**
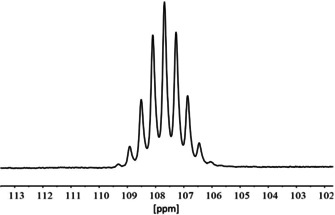
^27^Al NMR spectrum of [EtP_4_H][Al(C_2_F_5_)_4_] in Et_2_O with acetone‐[d_6_] as external standard in a capillary.

**Figure 2:**
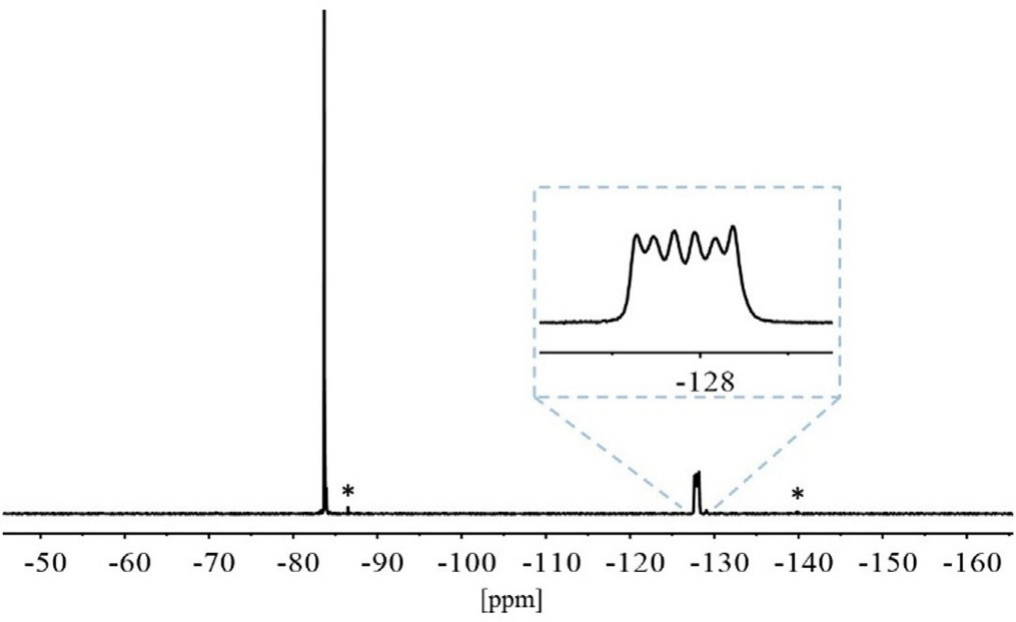
^19^F NMR spectrum of [PPh_4_][Al(C_2_F_5_)_4_] in Et_2_O with acetone‐[d_6_] as external standard in a capillary. *Signals for HC_2_F_5_.

The identity of the [Al(C_2_F_5_)_4_]^−^ ion is also evidenced by accurate mass measurement of the parent peak of [Al(C_2_F_5_)_4_]^−^ at *m*/*z=*502.9505 (calcd 502.95016 for C_8_F_20_Al^−^). Fragmentation occurs by extrusion of C_2_F_4_ units, revealing characteristic peaks at *m*/*z* (%)=402.95 (73) [Al(C_2_F_5_)_3_F]^−^, 302.96 (11) [Al(C_2_F_5_)_2_F_2_]^−^, 202.97 (2) [Al(C_2_F_5_)F_3_]^−^ and 102.97 (<1) [AlF_4_]^−^.

Single crystals of [PPh_4_][Al(C_2_F_5_)_4_] suitable for X‐ray diffraction were grown by diffusion of *n*‐hexane into a dichloromethane solution of the salt at −40 °C. Just like the higher homologue [PPh_4_][Ga(C_2_F_5_)_4_],^[15]^ [PPh_4_][Al(C_2_F_5_)_4_] crystallizes in the tetragonal space group *I*4_1_/*a* with 4 formula units per unit cell, meaning both ions exhibit *S*_4_ symmetry (Figure 3).^[34]^ The tetrahedral coordination sphere around the aluminum atom is slightly distorted as evident from the C1‐Al‐C1^1^ and C1‐Al‐C1^2^ angles of 105.4(1)° and 111.6(1)°. The Al−C1 bond length of 204.0(2) pm is well comparable to those of the anion [Al(C_2_H_5_)_4_]^−^ in alkali metal salts (201–203 pm)^[35]^ and to [Al(C_6_F_5_)_4_]^−^ (200–203 pm).^[36]^

**Figure 3:**
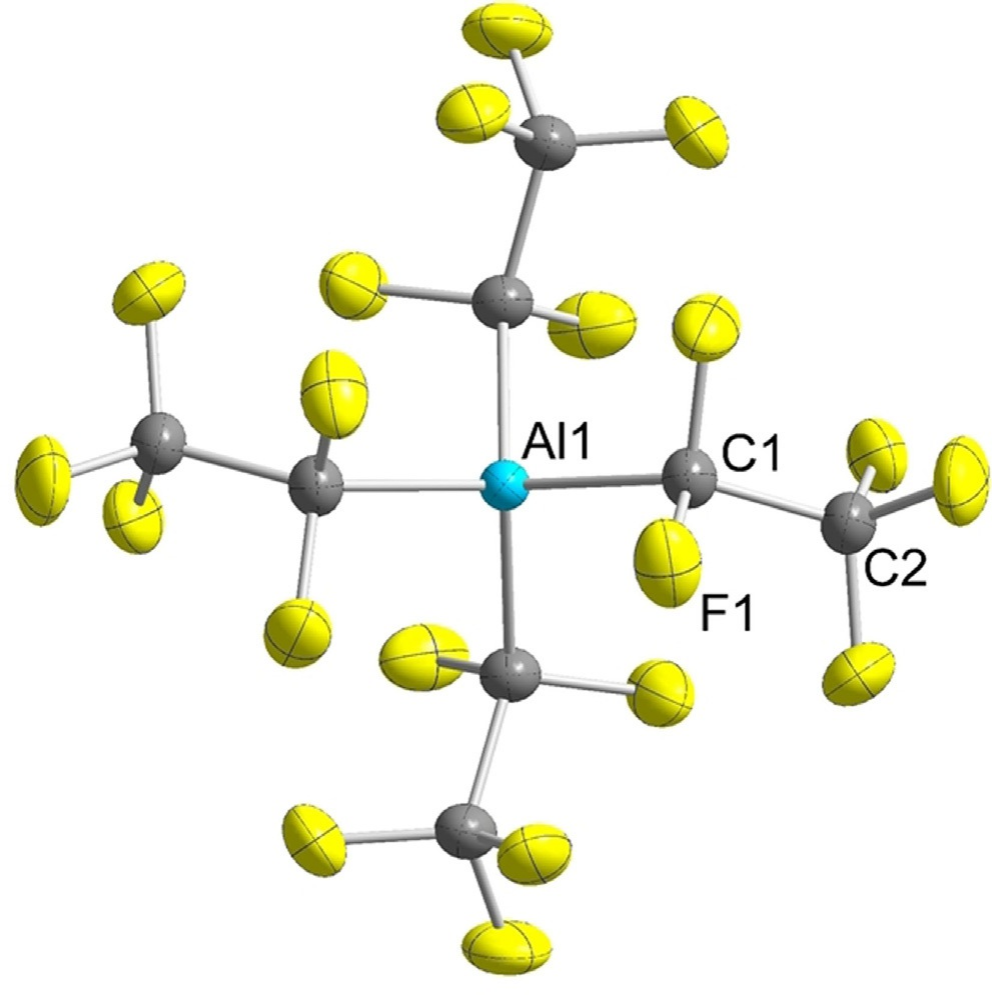
Molecular structure of the anion in [PPh_4_][Al(C_2_F_5_)_4_]. The cation is omitted for clarity. Thermal ellipsoids are set at 50 % probability. Selected bond lengths [pm] and angles [°]: Al1−C1 204.0(2), C1−F 137.9(2)–138.8(2), C2−F 132.8(2)–134.2(3), C1−C2 152.1(3); C1‐Al‐C1^1^ 105.4(1), C1‐Al‐C1^2^ 111.6(1), Al1‐C1‐C2 120.3(2). Symmetry codes: 1=1−X, 3/2−Y, +Z; 2=5/4−Y, 1/4+X, 1/4−Z.

We presented a convenient synthesis of [Al(C_2_F_5_)_4_]^−^ salts by reaction of Si(C_2_F_5_)_3_CH_3_ with Li[AlH_4_]. Subsequent cation exchange with [PPh_4_]Cl, [NBu_4_]Cl and [PNP]Cl affords the corresponding [Al(C_2_F_5_)_4_]^−^ salts. Utilizing the weakly coordinating phosphazenium cation [EtP_4_H]^+^, it was possible to increase the yield up to 85 % and to enhance the thermal stability. The salt decomposes above 110 °C. To the best of our knowledge, these salts represent the first examples of perfluoroalkyl aluminum derivatives that were isolated and fully characterized.

## Experimental Section

**Materials and apparatus**: All reactions were performed in the absence of water and air by use of standard *Schlenk* techniques. Chemicals were obtained from commercial sources and used without further purification. NMR spectra were either recorded on a Bruker Avance III 300 or Bruker Avance III 500 HD in the indicated solvent with acetone‐[d_6_] as lock substance in a capillary. Positive shifts are downfield from the external standards (TMS for ^1^H and ^13^C, H_3_PO_4_ for ^31^P, CCl_3_F for ^19^F and Al(NO_3_)_3_ for ^27^Al). IR spectroscopic measurements were performed on a Bruker Alpha‐FT‐IR spectrometer with a diamond crystal. ESI mass spectra were recorded using a *ZQ2000* single quadrupole mass spectrometer (Waters, Manchester, UK) equipped with an ESI source (3.5 kV spray voltage). Accurate mass nano‐ESI measurements were performed using a Q‐IMS‐TOF mass spectrometer Synapt G2Si (Waters Limited, Manchester, UK) in resolution mode, interfaced to a nano‐ESI ion source. The melting point was measured on a Mettler Toledo Mp70 Melting Point System. C, H, N analysis was conducted with a HEKAtech Euro EA 3000 apparatus. SCXRD was performed on a Rigaku Supernova diffractometer.

**[EtP_4_H][Al(C_2_F_5_)_4_]**: A sample of Si(C_2_F_5_)_3_CH_3_ (2.84 g, 7.10 mmol) was dissolved in Et_2_O (10 mL) and combined with a 1 m solution of Li[AlH_4_] in Et_2_O (1.26 g, 1.77 mmol) at rt. The reaction mixture was evaporated to dryness to give a colorless solid. The residue was redissolved in Et_2_O and treated with [EtP_4_H]Cl (1.65 g, 1.79 mmol). The reaction mixture was stirred for 17 h at rt and the precipitate was filtered off. [EtP_4_H][Al(C_2_F_5_)_4_] was recrystallized from the filtrate and isolated as a colorless solid in an 85 % (2.09 g, 1.50 mmol) yield.

m.p. 112 °C (only decomposition); ^**19**^ **F NMR** (282.4 MHz, Et_2_O, rt): *δ*=−83.5 (s, 12 F, CF_3_); −127.9 ppm (six‐line multiplet, ^2^ *J*(^19^F, ^27^Al) ≈32 Hz, 8 F, CF_2_); ^**27**^ **Al NMR** (78.2 MHz, Et_2_O, rt): *δ*=107.7 ppm (non, ^2^ *J*(^19^F, ^27^Al) ≈32 Hz, Al); ^**31**^ **P NMR** (202.5 MHz, Et_2_O, rt): *δ*=8.4 (dm, ^2^ *J*(^31^P, ^31^P)=71, ^3^ *J*(^1^H, ^31^P)=10 Hz, 3 P, (Et_2_N)_3_P), −33.0 ppm (qd, ^2^ *J*(^31^P, ^31^P)=71, ^2^ *J*(^1^H, ^31^P)=7 Hz, 1 P, P=NH); ^**1**^ **H NMR** (500.2 MHz, Et_2_O, rt): *δ*=3.44 (m, 36 H, (CH_3_C**H**_2_)_2_NP), 2.37 (d, ^2^ *J*(^1^H, ^31^P)=7 Hz, 1 H, NH), 1.58 (s, 9 H, P=NC(CH_3_)_3_), 1.39 ppm (m, 54 H, (C**H**_3_CH_2_)_2_NP); ^**13**^ **C{^1^H} APT NMR** (125.8 MHz, Et_2_O, rt): *δ*=50.8 (d, ^2^ *J*(^13^C, ^31^P)=4 Hz, P=N**C**(CH_3_)_3_), 39.3 (d, ^2^ *J*(^13^C, ^31^P)=6 Hz, (CH_3_ **C**H_2_)_2_NP), 31.3 (d, ^3^ *J*(^13^C, ^31^P)=5 Hz, P=NC(**C**H_3_)_3_), 13.0 ppm (d, ^3^ *J*(^13^C, ^31^P)=4 Hz, (**C**H_3_CH_2_)_2_NP); **IR** (ATR): $\tilde{\nu }$ =2970 (w), 2934 (w), 2872 (w), 1462 (w), 1379 (w), 1351 (w), 1268 (m), 1199 (m), 1173 (vs), 1096 (m), 1017 (vs), 938 (s), 848 (w), 792 (m), 736 (m), 702 (s), 612 (m), 534 (m), 510 (s), 438 cm^−1^ (m); **MS** (ESI, pos., THF): *m*/*z* (%): 886.9 (100) [EtP_4_H]^+^; **MS** (ESI, neg., THF): *m*/*z* (%): 503.1 (6) [Al(C_2_F_5_)_4_]^−^; **elemental analysis** calcd (%) for C_48_H_100_AlF_20_N_13_P_4_: C 41.47, H 7.25, N 13.10; found: C 41.59, H 7.56, N 13.33.

**[PPh_4_][Al(C_2_F_5_)_4_]**: Analogously to the synthesis of [EtP_4_H][Al(C_2_F_5_)_4_], the reaction of Si(C_2_F_5_)_3_CH_3_ (8.28 g, 20.7 mmol), Li[AlH_4_] (6.26 g, 8.75 mmol), and [PPh_4_]Cl (3.30 g, 8.80 mmol) afforded [PPh_4_][Al(C_2_F_5_)_4_] in a 63 % yield (4.62 g, 5.48 mmol); ^**19**^ **F NMR** (282.4 MHz, Et_2_O, rt): *δ*=−83.5 (s, 12 F, CF_3_); −127.7 ppm (six‐line multiplet, ^2^ *J*(^19^F, ^27^Al) ≈32 Hz, 8 F, CF_2_); ^**27**^ **Al NMR** (78.2 MHz, Et_2_O, rt): *δ*=107.7 ppm (non, ^2^ *J*(^19^F, ^27^Al) ≈32 Hz, Al); ^**31**^ **P{^1^H}** **NMR** (121.5 MHz, Et_2_O, rt): *δ*=23.9 ppm (s, ^1^ *J*(^31^P, ^13^C)=89 Hz, P); ^**1**^ **H NMR** (500.2 MHz, Et_2_O, rt): *δ*=8.67 (m, 4 H, *para*‐CH), 8.51 (m, 8 H, *meta*‐CH), 8.40 ppm (m, 8 H, *ortho*‐CH); ^**13**^ **C{^1^H} APT NMR** (125.8 MHz, Et_2_O, rt): 136.3 (d, ^4^ *J*(^13^C, ^31^P)=3 Hz, *para*‐CH), 135.0 (d, ^3^ *J*(^13^C, ^31^P)=11 Hz, *meta*‐CH), 131.3 (d, ^2^ *J*(^13^C, ^31^P)=13 Hz, *ortho*‐CH), 118.4 ppm (d, ^1^ *J*(^13^C, ^31^P)=89 Hz, *ipso*‐C); **IR** (ATR): $\tilde{\nu }$ =1587 (w), 1485 (w), 1438 (w), 1306 (m), 1286 (w), 1176 (s), 1097 (s), 1008 (m), 996 (m), 928 (m), 847 (w), 753 (m), 722 (s), 689 (s), 634 (w), 584 (w), 526 (vs), 437 cm^−1^ (s); **HRMS** (ESI, neg.): *m*/*z* calcd for C_8_F_20_Al^−^: 502.95016; found: 502.9505. fragmentation: *m*/*z* (%): 402.95 (73) [Al(C_2_F_5_)_3_F]^−^, 302.96 (11) [Al(C_2_F_5_)_2_F_2_]^−^, 202.97 (2) [Al(C_2_F_5_)F_3_]^−^, 102.97 (<1) [AlF_4_]^−^.

## Conflict of interest

The authors declare no conflict of interest.

## Supporting information

As a service to our authors and readers, this journal provides supporting information supplied by the authors. Such materials are peer reviewed and may be re‐organized for online delivery, but are not copy‐edited or typeset. Technical support issues arising from supporting information (other than missing files) should be addressed to the authors.

Supplementary
