# Supplementary material for: Synthesis and Characterization of Tetrakis(pentafluoroethyl)aluminate
Source: Chemistry. 2020 Sep 17;26(60):13611–4. doi: 10.1002/chem.202000668 (PMC7693355; doi:10.1002/chem.202000668)
Supplement: Supplementary file 1 — Supplementary [file CHEM-26-13611-s001.pdf]

Chemistry–A European Journal

Supporting Information

Synthesis and Characterization of Tetrakis(pentafluoroethyl)aluminate

Natalia Tiessen, Beate Neumann, Hans-Georg Stammler, and Berthold Hoge*[a]

NMR spectra of $[PPh_4][Al(C_2F_5)_4]$

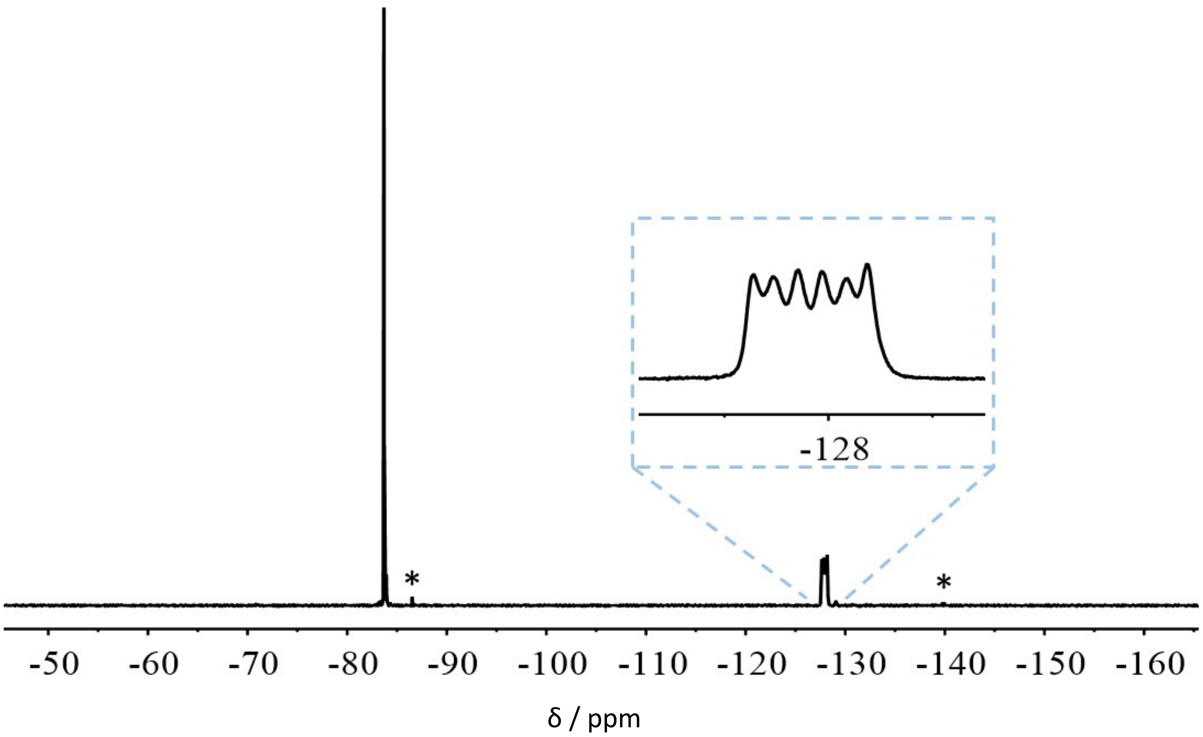

Figure S1: ^{19}F NMR spectrum of $[PPh_4][Al(C_2F_5)_4]$ in Et_2O with acetone-[d_6] as external standard.
* Signals for HC_2F_5.

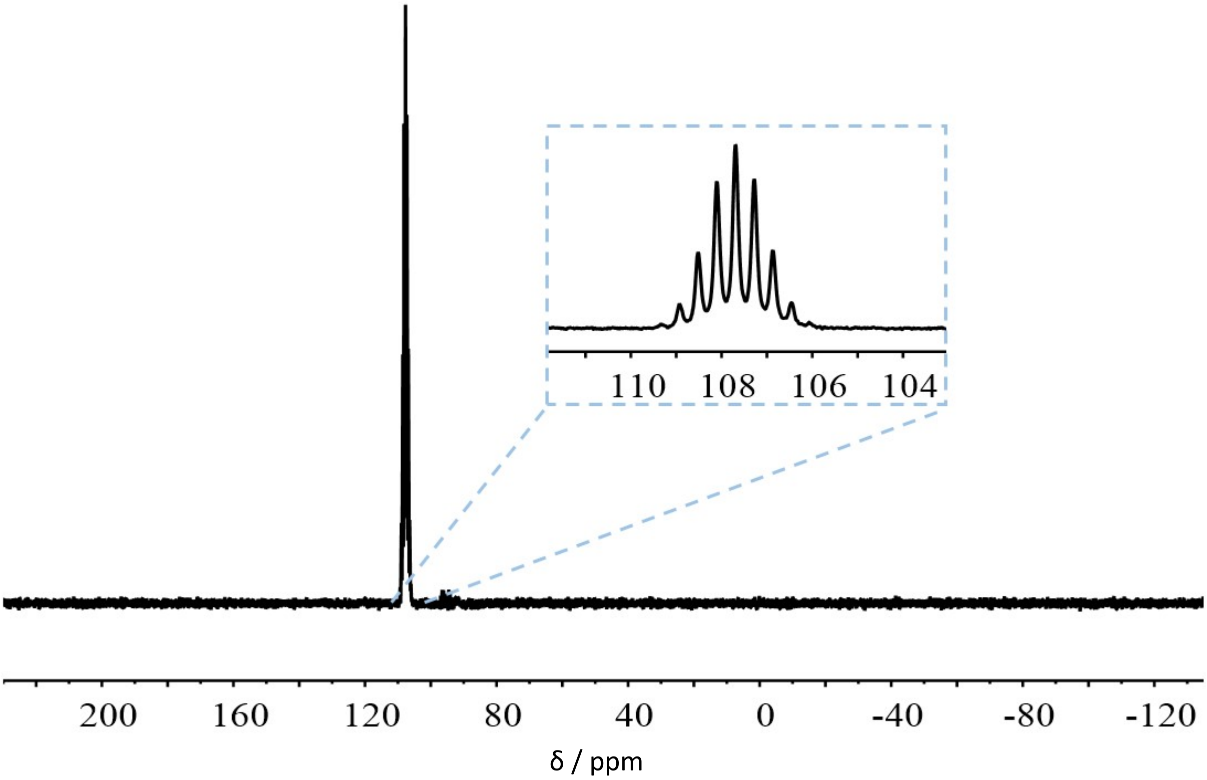

Figure S2: ^{27}Al NMR spectrum of $[PPh_4][Al(C_2F_5)_4]$ in Et_2O with acetone-[d_6] as external standard.

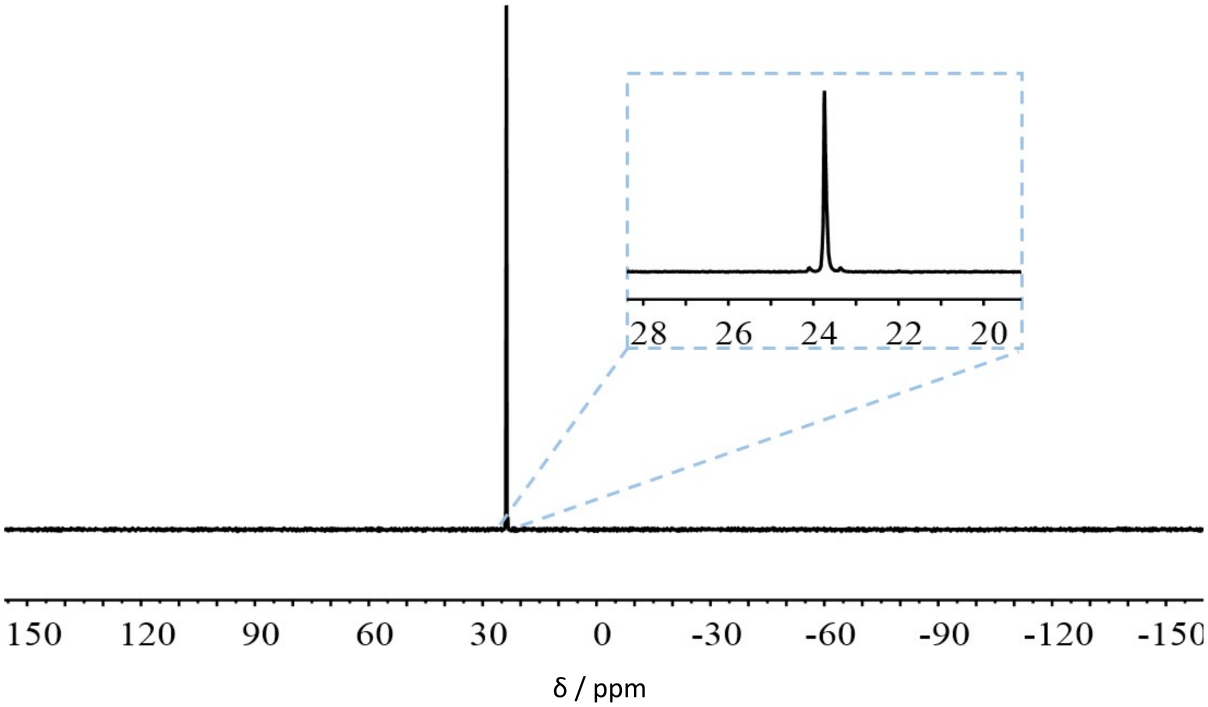

Figure S3: ^{31}P NMR spectrum of $[PPh_4][Al(C_2F_5)_4]$ in Et_2O with acetone-[d_6] as external standard.

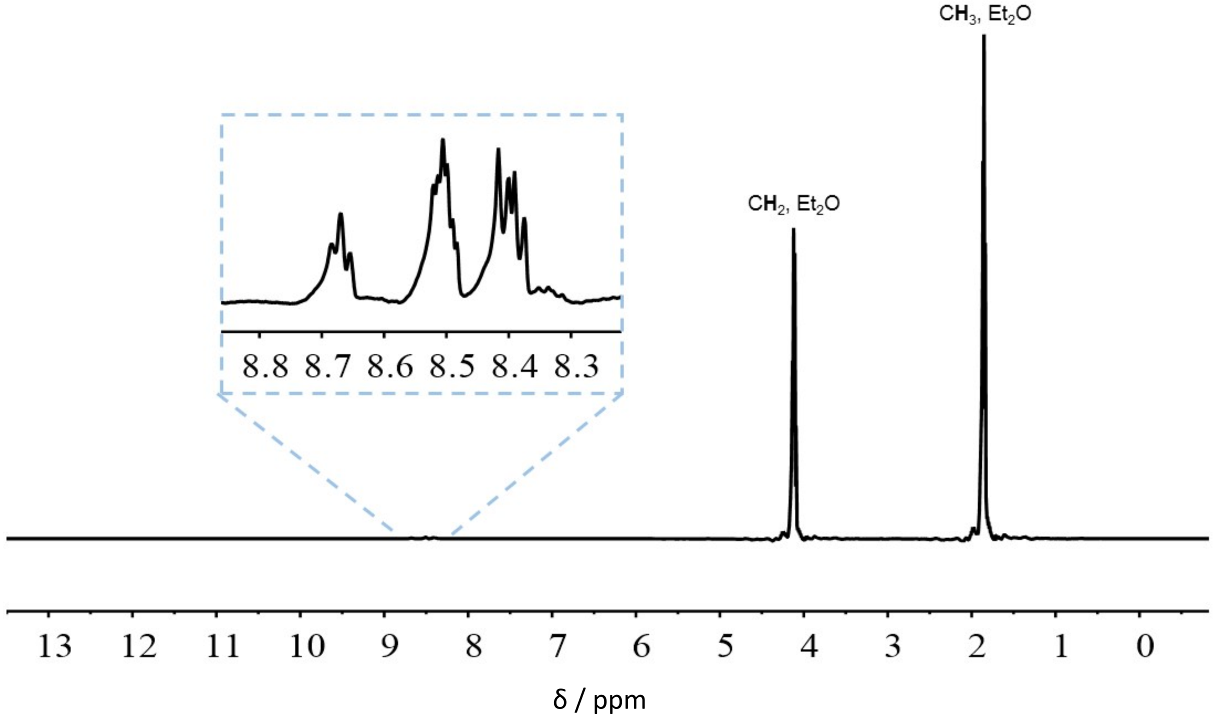

Figure S4: ^{1}H NMR spectrum of $[PPh_4][Al(C_2F_5)_4]$ in Et_2O with acetone-[d_6] as external standard.

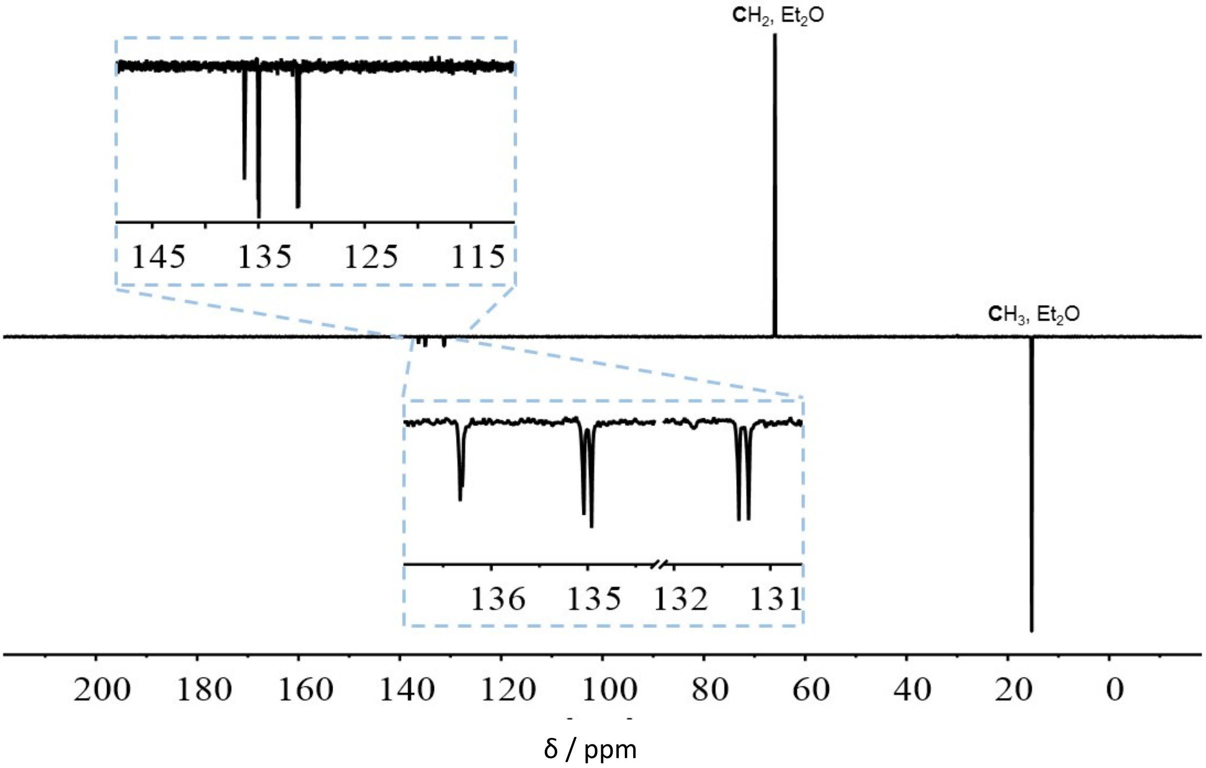

Figure S5: $^{13}C\{^1H\}$ APT NMR spectrum of $[PPh_4][Al(C_2F_5)_4]$ in Et_2O with acetone-[d_6] as external standard.

NMR spectra of [EtP$_4$H][Al(C$_2$F$_5$)$_4$]

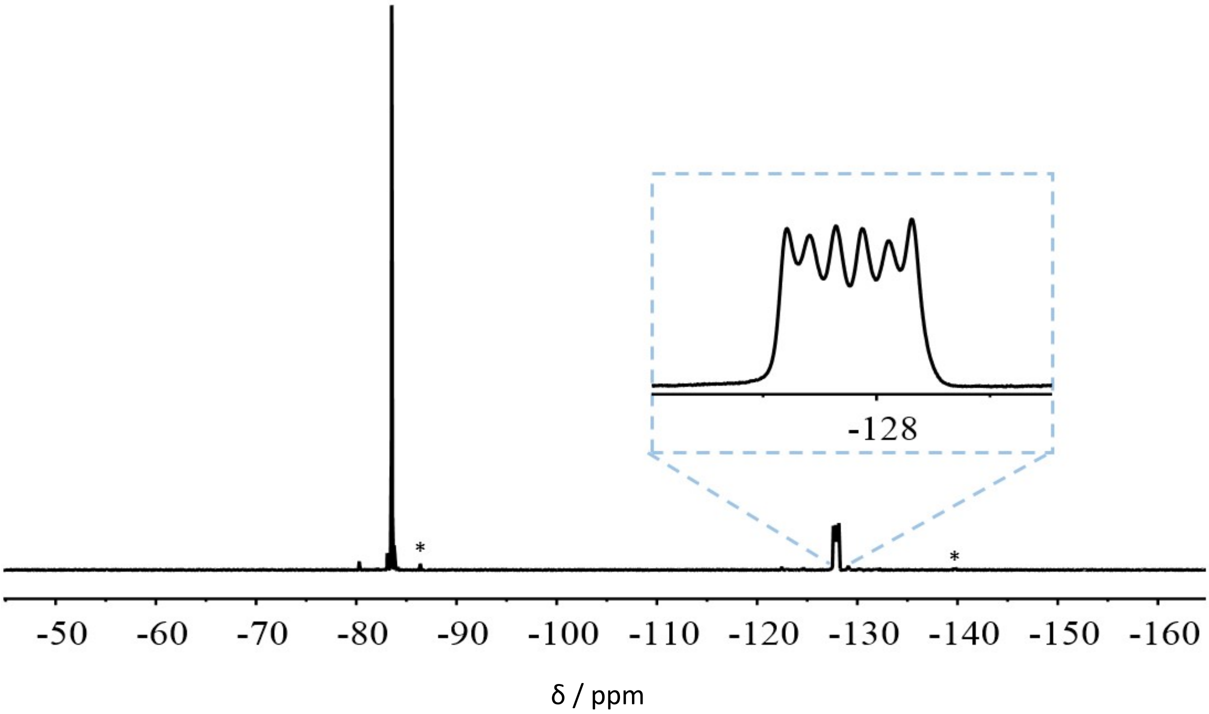

Figure S6: ^{19}F NMR spectrum of [EtP$_4$H][Al(C$_2$F$_5$)$_4$] in Et$_2$O with acetone-[d$_6$] as external standard.
* Signals for HC$_2$F$_5$.

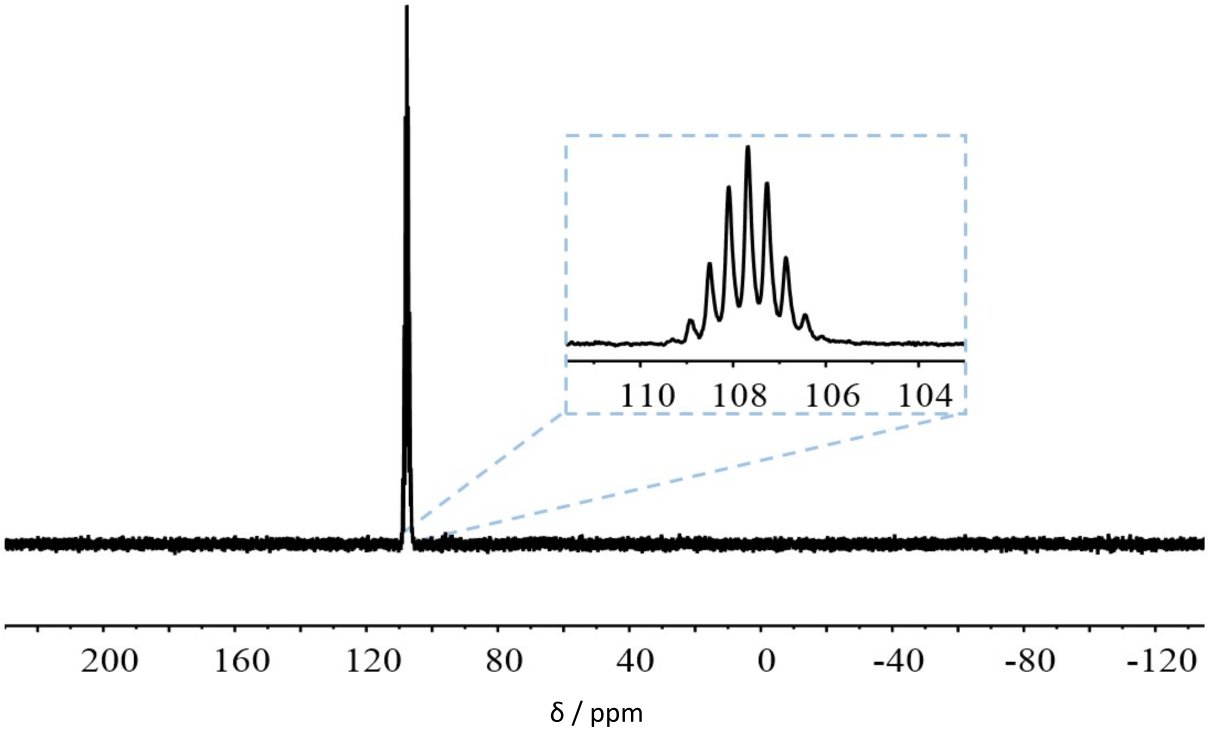

Figure S7: ^{27}Al NMR spectrum of [EtP$_4$H][Al(C$_2$F$_5$)$_4$] in Et$_2$O with acetone-[d$_6$] as external standard.

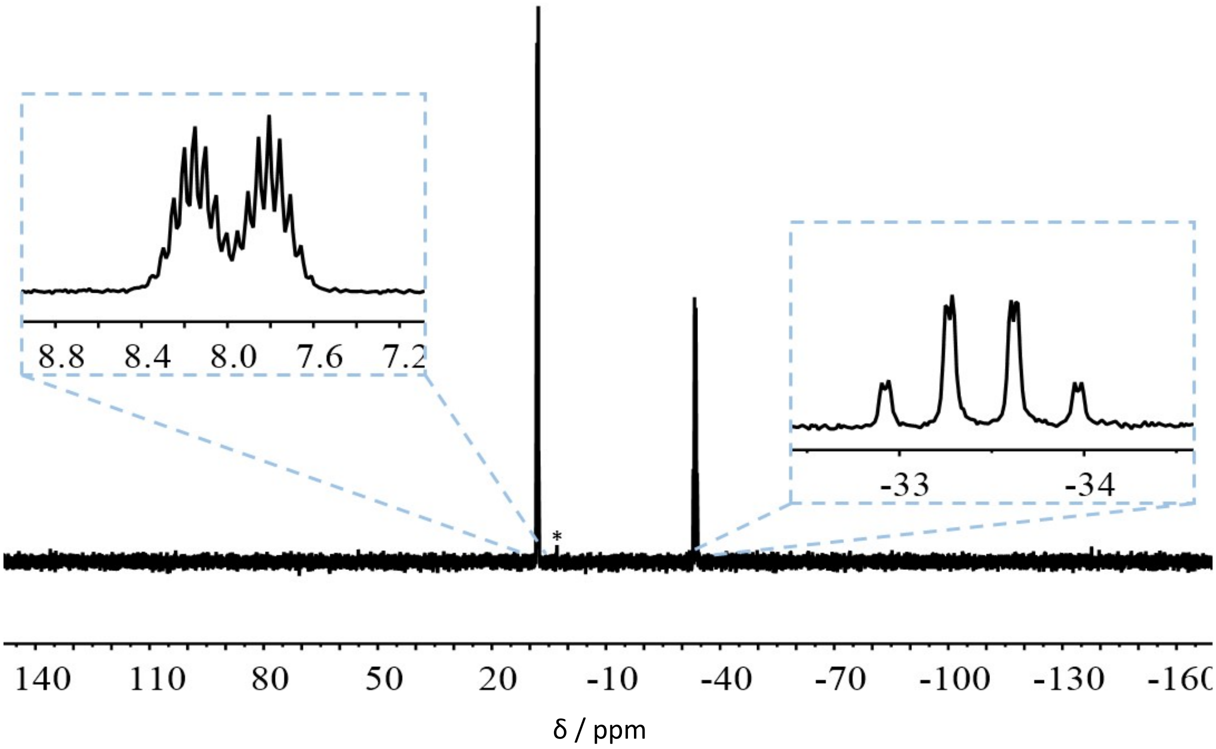

Figure S8: ^{31}P NMR spectrum of $[EtP_4H][Al(C_2F_5)_4]$ in Et_2O with acetone-[d_6] as external standard.
* Signal for $OP(OMe)_3$ as external standard in a capillary.

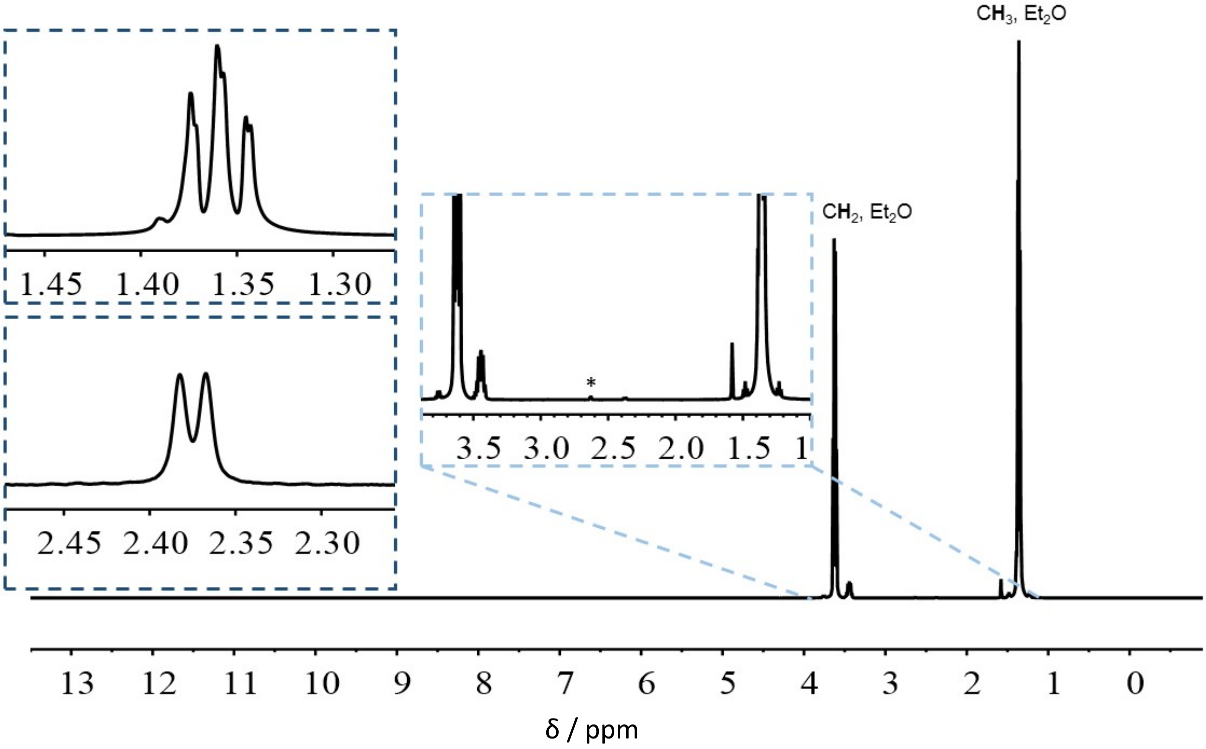

Figure S9: ^{1}H NMR spectrum of $[EtP_4H][Al(C_2F_5)_4]$ in Et_2O with acetone-[d_6] (*) as external standard.

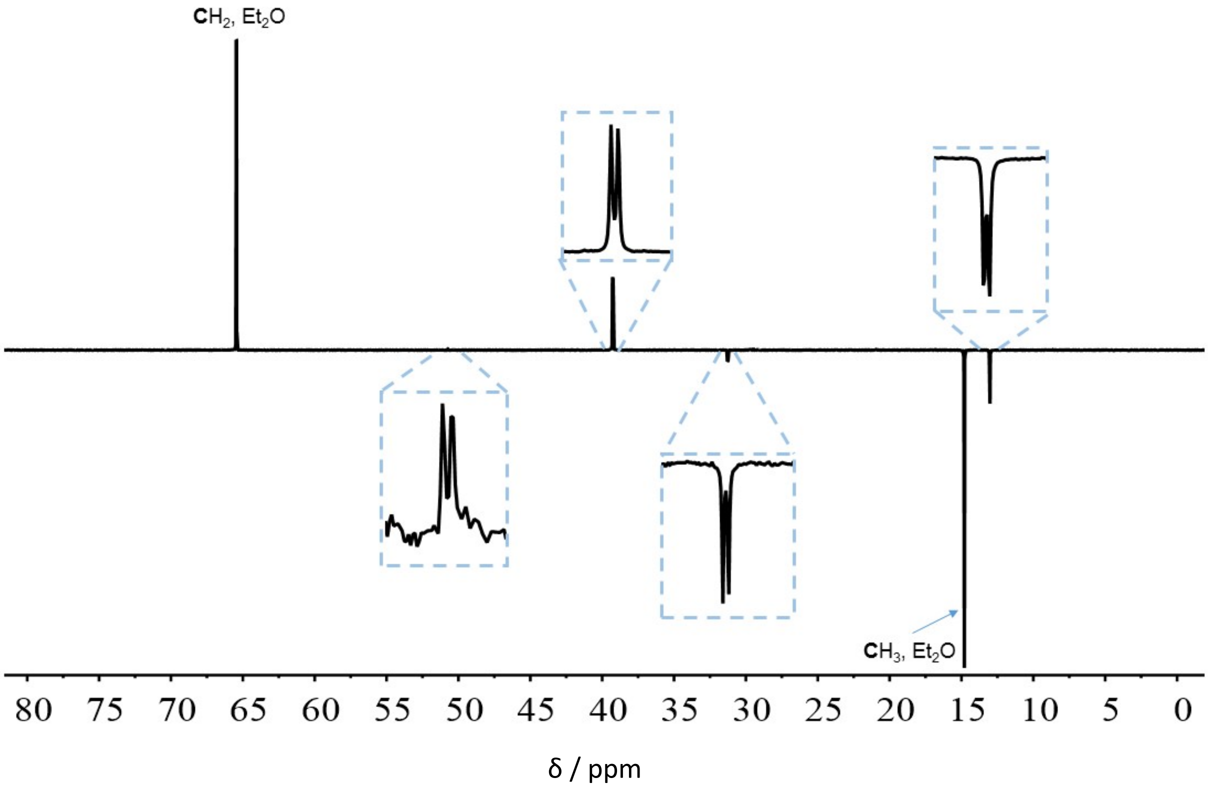

Figure S10: $^{13}C\{^{1}H\}$ APT NMR spectrum of $[EtP_4H][Al(C_2F_5)_4]$ in Et_2O with acetone-$[d_6]$ as external standard.
